# Physicochemical and Antimicrobial Characterization of Nanobubbles Reveals Physical Disruption is the Primary Mode of Biofilm Inactivation

**DOI:** 10.1021/acsestwater.6c00252

**Published:** 2026-06-01

**Authors:** Naomi Northage, Matjaž Gomilšek, Martina Modic, Damjan Vengust, Andrej Zorko, Uroš Cvelbar, James L. Walsh

**Affiliations:** † 61790 Jožef Stefan Institute, Ljubljana SI-1000, Slovenia; ‡ Faculty of Mathematics and Physics, University of Ljubljana, Ljubljana SI-1000, Slovenia; § York Plasma Institute, School of Physics, Engineering & Technology, 8748 University of York, York YO10 5DD, U.K.

**Keywords:** nanobubbles, ultrafine bubbles, reactive oxygen species, hydroxyl radicals, biofilms, decontamination, oxidative stress

## Abstract

Biofilm-associated contamination represents a persistent and costly challenge across environmental systems, causing reduced efficacy of disinfectants. Recently, nanobubbles (NBs) have shown promise for biofilm decontamination; yet, their underpinning mode of action remains a topic of debate. In this study, the interaction of air-generated NBs with *Escherichia coli* and *Staphylococcus aureus* biofilms was investigated. NBs were generated using a venturi nozzle and characterized using Nanoparticle Tracking Analysis, revealing a NB density of 5.66 × 10^8^ particles/mL and a mean diameter of 84 nm. Application of NB solution to microbial biofilms resulted in a 2.16 log reduction for *E. coli* and 1.52 log reduction for *S. aureus*, along with visible morphological changes such as cell collapse, wrinkling, and matrix disruption. ESR spin trapping confirmed hydroxyl radical formation, but intracellular ROS and lipid peroxidation levels were minimal and, in some cases, not significantly different from Milli-Q water controls. After 28 days, NBs remained present and continued to demonstrate antimicrobial activity, biofilm disruption, and some ROS activity. These findings indicate that although hydroxyl radicals are generated, oxidative stress is not the dominant antimicrobial mechanism under the examined conditions, suggesting physical biofilm disruption is the primary mode of action.

## Graphical abstract

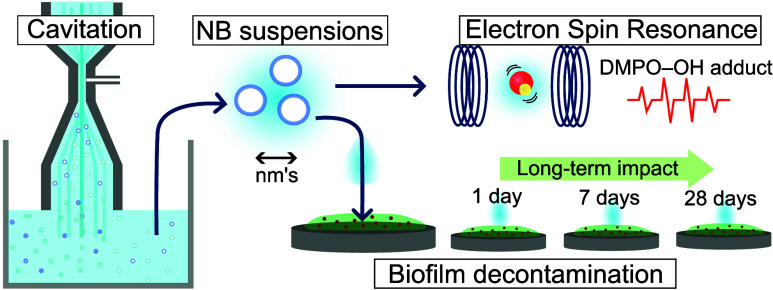

## Introduction

Biofilm-associated contamination represents a persistent and costly challenge across environmental systems due to reduced efficacy of disinfection processes leading to biofouling and long-term microbial persistence in water systems.^1,2^ Microorganisms such as *Escherichia coli* and *Staphylococcus aureus* are widely used as representative Gram-negative and Gram-positive model organisms, respectively, for studying biofilm behavior due to their presence in environmental and industrial contexts.^3–5^ These biofilm-forming microorganisms create complex structures of cells adhered to each other and to surfaces within a matrix of extracellular polymeric substances (EPS).^6^ When biofilm formation occurs, there is increased resistance to cleaning and disinfection methods, causing significant issues in water treatment settings.^7–10^ In water treatment and water systems, biofilms can form on pipe interiors, filter media, membrane surfaces, storage tanks, sedimentation basins, and other components where moisture and nutrients facilitate microbial attachment.^11^ These pathogens can detach from pipe biofilms into treated water, causing risk to consumers and complicating compliance with microbial water quality standards.^12^

There is increasing recognition that traditional chemical disinfection methods, such as chlorine, chloramine, hydrogen peroxide, and peracetic acid, pose a number of environmental and occupational health challenges. These agents act nonspecifically against multiple cellular targets, including the cell wall or outer membrane, the cytoplasmic membrane, functional and structural proteins, DNA, RNA, and other cytosolic components; however, their broad reactivity is associated with the generation of hazardous byproducts, increased corrosivity, and risks of acute and chronic exposure for workers, as well as potential adverse impacts on surrounding ecosystems.^9,13,14^ For example, chlorine residuals are toxic to aquatic life and may require dechlorination, while all forms of chlorine are highly corrosive and toxic; thus, storage, shipping, and handling pose a risk requiring increased safety regulations.^15^ In addition, chlorine can oxidize various types of organic matter in wastewater, which can lead to the formation of more hazardous compounds, e.g., trihalomethanes.^16^ Chlorine is harmful to staff and can cause immediate health issues such as respiratory irritation, eye and skin burns, and, in high concentrations, suffocation.^17^ Long-term exposure can lead to chronic lung problems such as asthma and bronchitis and in some cases may cause skin issues from repeated contact.^17^ Peracetic acid is corrosive and irritating to the eyes, mucous membranes of the respiratory tract, and skin.^18^ It can cause lacrimation, extreme discomfort, and irritation to the upper respiratory tract in humans after exposure to concentrations as low as 15.6 mg of peracetic acid/m^3^ (5 ppm) for only 3 min.^18^ It can also cause fixation of biofilms on surfaces, further complicating disinfection.^19^

Recently, nanobubbles (NBs), otherwise known as ultrafine bubbles, have gained interest in various fields, including wastewater treatment, because of their unique physical and chemical properties.^20,21^ NBs are less than 1000 nm in diameter and are characterized by a large gas/liquid contact area, extended stability in solution, and a negative surface charge.^22^ Furthermore, it has been suggested that free radicals may be generated during bubble shrinkage or collapse.^20,22^ In wastewater treatment, they have been used for their ability to remove micropollutants and enhance water purification processes.^20,21^ A recent study showed that oxygen micro/nanobubbles (MNBs) significantly inhibited biofilm formation in drinking water systems, reducing biofilm dry weight by up to 77.87% and removing 87.9% of total organic carbon.^23^ Despite such promising results, debate within the literature is ongoing regarding the generation of reactive oxygen species (ROS) from NB collapse and whether it is in sufficient quantities to contribute to the antimicrobial effects observed. The mechanism of the antimicrobial action of NBs, possibly involving physical disruption, oxidative stress via ROS, gas exchange, or surface interactions, is still not fully understood.^24^ Antimicrobial activity is often inferred from physicochemical characterization alone, without directly correlating ROS generation to cellular oxidative stress responses; as a result, the relative contribution of oxidative versus nonoxidative mechanisms remains unclear.

Within this study, we directly address this mechanistic uncertainty by correlating detailed physicochemical characterization of air-generated NBs, including ESR-based hydroxyl radical detection, with biological endpoints that report on oxidative stress, membrane integrity, and biofilm viability. Biofilms of two bacterial strains, *E. coli* and *S. aureus*, representing Gram-negative and Gram-positive bacteria, were formed on medical-grade stainless steel. The physicochemical properties of NBs, including pH, conductivity, size, zeta potential, and hydroxyl radical (^•^OH) generation, were characterized. Electron spin resonance (ESR) spectroscopy was used to detect free radicals. The effect of NBs on biofilms was assessed using standard culture methods, scanning electron microscopy (SEM), and live/dead staining to evaluate structural integrity and cell viability. Oxidative stress was evaluated using lipid peroxidation, intracellular ROS, and reactive nitrogen species (RNS) assays. Finally, NB stability over time, ROS evolution, and the long-term antimicrobial efficacy against biofilms were explored.

## Materials and Methods

### Nanobubble Generation

The NB suspensions used within this study were produced using a NB generator (OKE-MB60 mL-PT1/8.MM, OK Engineering, Japan). As depicted in Figure 1, 500 mL of Milli-Q water (MQ water, OmniaTap, Stakpure GmbH) was circulated through the NB generator using a peristaltic pump operating at a flow rate of 60 mL/min, as per the manufacturer’s instructions. The water was circulated for 5-, 10-, 15-, 20-, 25-, and 30-min and ambient air was used for NB generation.

**Figure 1:**
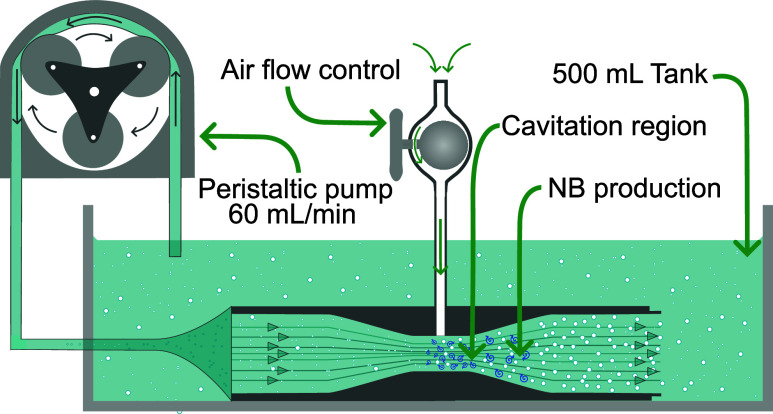
Schematic of nanobubble (NB) generator used to produce NB suspensions. Ambient air was used as the gas source, and Milli-Q water was circulated at a flow rate of 60 mL/min.

### Characterization of Nanobubbles

The size and concentration of NBs were measured using nanoparticle tracking analysis (NTA, NanoSight NS300, Malvern Instruments, Worcestershire, UK) and analyzed using NS XPLORER v1.1.0.6 (Malvern Instruments, Worcestershire, UK). Each sample was loaded into the machine and passed through the system using a syringe pump at a flow rate of 3.0 μL/min. The temperature and pH were measured using a pH meter (PC 52+ DHS, XS Instruments, Italy). The conductivity and zeta potential of the suspension were measured using the Zetasizer Ultra (Malvern Instruments, Worcestershire, UK). In addition, terephthalic acid (TA) was used for the detection of hydroxyl radicals in the NB suspension. TA reacts with hydroxyl radicals to form 2-hydroxyterephthalic acid (TAOH), which can be detected using fluorescence spectroscopy at λ_ex/em_ = 310/425 nm.^25^

### Hydroxyl Radical Determination Using ESR

ESR spectroscopy was used to detect hydroxyl radical generation. DMPO is a commonly used spin trap that reacts with O-, N-, S-, and C-centered radicals allowing their characterization, particularly of hydroxyl radicals and superoxide anions, when used in association with ESR.^26^ The NB samples were mixed with 300 mM 5,5-dimethyl-1-pyrroline *n*-oxide (DMPO) and the sample transferred to a quartz tube (effective volume 100 μL) and the spectra measured using a Bruker Elexsys E500 ESR spectrometer operating at 9.39 GHz, equipped with a Bruker ER 049X microwave bridge, and a Superhigh-Q resonator ER 4122 SHQ. The samples were frozen and cooled to 250 K, where they were measured with a 0.1 mT modulation amplitude. An Oxford Cryogenics continuous-flow liquid helium cryogenic system ensured temperature stability better than 0.1 K.

In all presented ESR fits, the applied fields *B* were normalized to a common microwave frequency of 9.393002 GHz, and a quintic background contribution was subtracted in the fits. The sample signal was then fit using EasySpin simulations of DMPO-OH and triplet signals (using the *garlic* method for simulations of fast-motion continuous-wave ESR spectra). Using a global fit of all the data sets, accurate anisotropic parameters of these signals were obtained, presented in Table S1. The parameters of the signals were broadly consistent with their isotropic estimates from the literature.^27^ These signal parameters were kept fixed in the final fits, with only their individual amplitudes and a common overall *g*-factor rescaling (close to 1) remaining as the only free fit parameters, an approach that yielded an excellent fit quality. Integration of the simulated components was used to define the DMPO-OH fraction (*f*) as the ratio of the DMPO-OH contribution to the total ESR signal intensity. Continuous ESR measurements were performed by adding DMPO immediately after NB preparation and monitoring spectra over 24 h at 250 K, while long-term radical activity was assessed by storing samples for defined periods prior to the addition of DMPO and ESR analysis.

### Bacterial Strains and Growth Conditions

Two bacterial strains were used in this study, *E. coli* (ATCC No. 25922) and *S. aureus* (ATCC No. 25923). These strains were chosen as examples of both Gram-negative and Gram-positive bacterial strains, respectively. Both bacterial strains were maintained on Tryptic soy agar (TSA), and single colonies from each agar plate were used to inoculate 5 mL of Tryptic soy broth (TSB). The inoculated media was left to incubate at 37 °C and 120 rpm, shaking for 24 h. The concentration of each inoculum was adjusted to 1 × 10^6^ colony forming units (CFU)/mL by broth dilution before use.

### Biofilm Formation on Stainless Steel Coupons

Single species *E. coli* and *S. aureus* biofilms were formed on medical-grade Type 304 stainless steel coupons (No. 2b finish, 2.54 cm × 7.62 cm × 0.081 cm, Biosurface, Bozeman, MT, USA). Prior to use, coupons were autoclaved at 121 °C for 20 min, soaked with 70% ethanol, air-dried and reautoclaved at 121 °C for 20 min. Overnight *E. coli* and *S. aureus* cultures were adjusted to a final concentration of 1 × 10^6^ CFU/mL using broth dilution with fresh TSB. The stainless steel coupons were placed in wells of a 24-well plate with 1 mL of the inoculum placed in each well. Coupons were left to incubate at 37 °C and 120 rpm shaking for 24 h, at which point biofilm formation and disinfection were assessed.

### Impact of Nanobubbles on the Biofilm Structure and Viability

Following biofilm formation, the coupons were rinsed with sterile phosphate-buffered saline to remove planktonic cells. Coupons were then placed in a 24-well plate and exposed to 1 mL of NB suspension or MQ water for defined time periods under static conditions. The treatment solutions fully covered the coupon surface, ensuring a uniform exposure of the biofilm. After the defined treatment period, coupons were transferred to 5 mL of fresh TSB, vortexed, and agitated for 15 min to recover biofilm cells. Viable counts were then determined using the Miles and Misra plating method, following serial dilution of the recovered samples.

To examine morphological changes, biofilms were formed on the stainless steel coupons using the method described previously and then placed in fresh TSB (control), MQ water, or NBs for 1 h. In preparation for SEM analysis, coupons were rinsed and placed in a solution of 3% glutaraldehyde overnight to fix the biofilms. Next, dehydration was performed by placing the coupons in a series of ethanol solutions of increasing concentrations (50%, 60%, 70%, 80%, 90%, 95%, and 100% V/V twice) for 20 min, followed by solutions of hexamethyldisilane (HMDS) in ethanol (33%, 66%, and 100% V/V). Finally, the coupons were left to dry inside a fume hood, and 3 drops of HMDS were placed on them. The coupons were mounted in a SEM specimen holder using double-sided tape and analyzed in a SEM from Jeol (model JSM-7600F). All samples underwent identical fixation and dehydration procedures, indicating that the observed morphological differences arise from treatment effects rather than preparation artifacts.

Bacterial viability was assessed using the BacLight LIVE/DEAD Bacterial Viability Kit (Invitrogen, Molecular Probes, Carlsbad, CA, USA; L7012). 24 h single species biofilms were exposed to 1 mL of a NB suspension, MQ water, or TSB (control) and left to incubate for 1 h, the solution was subsequently removed, and the coupons washed two times with phosphate-buffered saline. A 1:1 ratio of SYTO9 green fluorescent nucleic acid stain and propidium iodide red fluorescent nucleic acid stain was prepared to label the live and dead bacteria, respectively. For each sample, 25 μL of the SYTO9 and propidium iodide stain was added to the coupon and left to incubate in the dark at room temperature for 15 min to allow the dye to bind to the bacteria. Cells were imaged under the microscope and samples were measured using a fluorescent microplate reader (λ_ex/em_ = 485/630 nm).

### Evaluation of Oxidative Stress

To evaluate the potential of NBs to induce oxidative stress in bacteria through hydroxyl radical generation, lipid peroxidation and intracellular ROS and RNS levels were measured. A lipid peroxidation assay (Image-iT Lipid Peroxidation Kit, Invitrogen) was used to stain the *E. coli* and *S. aureus* biofilms. First, biofilms were treated with MQ water or NBs for 1 h. A lipid peroxidation sensor was added at a final concentration of 10 μM and the coupons left to incubate for 30 min at 37 °C. Following treatment, the coupons were rinsed twice with PBS. The fluorescence was measured using the Texas Red and FITC filters and the ratio of their intensities calculated. Images were also taken with a fluorescence microscope, with images from Texas Red and FITC being merged and presented.

Intracellular ROS was measured using 2′,7′-dichlorodihydrofluorescein diacetate (H_2_DCFDA), a fluorogenic reagent, which becomes green when oxidation occurs within the cells. One loop of *E. coli* and *S. aureus* was used to inoculate 5 mL of TSB and left to incubate overnight. For evaluation of ROS in planktonic cells, 1 mL of each bacterial culture was centrifuged at 400*g* for 5 min and the cell pellet resuspended in 200 μL of fresh TSB. To detect the presence of ROS, 1 μL of H_2_DCFDA was added to the cells. Cultures were left to incubate at 37 °C, shaking for 30 min. The cells were centrifuged again using the same parameters and resuspended in TSB, MQ water, or NBs and then fluorescence measured at *E_x_*/*E*_m_: 504/529 nm. For evaluation of the presence of ROS in biofilm cells, biofilms were formed on stainless steel following the previously described procedure, treated with either MQ water or NBs for 1 h and then 5 μL of H_2_DCFDA was added and the fluorescence imaged using microscopy. Intracellular RNS was analyzed using an NO detection reagent (ab139473, Abcam, United States) and fluorescence measured at *E_x_*/*E*_m_: 650/670 nm.

### Long-Term Stability and Antimicrobial Efficacy of Nanobubbles

To assess the long-term stability and antimicrobial efficacy of NBs, the characteristics of NBs were measured for specified time periods of up to 1 month. Particle size, concentration, and stability were measured using NTA and ζ-potential measurements from the ZetaSizer. Hydroxyl radical determination as described previously was also carried out. Additionally, biofilm disinfection experiments, as previously described, were repeated using NBs stored for 1 day, 1 week, and 1 month to determine the impact of storage time on their effectiveness in disrupting *E. coli* and *S. aureus* biofilms.

### Data Analysis

Experiments were conducted with at least 3 biological repeats and/or 3 technical repeats. Results are presented as mean ± standard deviation. Statistical analysis was performed using GraphPad Prism 10.0 and OriginPro 2020b. Two-way ANOVA was applied where two independent variables were present (e.g., treatment and exposure time), followed by Tukey’s post hoc test for multiple comparisons. For single-factor comparisons, one-way ANOVA was used. A *p*-value < 0.05 was considered statistically significant. All fluorescence images presented within this work were adjusted using Image J in the same manner.

## Results and Discussion

### NB Characterization

Characterization of NBs was carried out by assessing the size, concentration, and physicochemical properties over different generation times at a constant liquid and air flow rate. NBs were created using the NB generator and sampled at 5-, 10-, 15-, 20-, 25-, and 30-min. The particle size distribution of NBs was measured using NTA and is displayed in Figure 2a–f. Supporting Movie 1 shows the Brownian motion of the NBs in suspension. A generation time of 10 min led to the highest concentration at 5.66 × 10^8^ particles/mL, after which the particle concentration began to decrease with increasing generation time to 4.32 × 10^8^, 4.41 × 10^8^, 2.97 × 10^8^, and 2.97 × 10^8^ particles/mL for 15-, 20-, 25-, and 30-min, respectively. The mean sizes of the NBs were 97-, 84-, 80-, 95-, 92-, and 85-nm for 5-, 10-, 15-, 20-, 25-, and 30-min, respectively. This is similar to that shown in other studies where the particle concentration increases with increasing generation time and then begins to decrease again after reaching maximum density.^28–30^ Zhou et al. showed that the concentration of micronanobubbles increased over time, reaching a maximum at 8 min, and then decreased at 10 min.^28^ In addition, the size initially decreased with longer generation times due to increased cavitation and bubble fragmentation, reaching a minimum NB diameter around 6–8 min. At longer times (8–10 min), the size increased again as high bubble density promoted coalescence and cluster formation.^28^ Consistent with these findings, Li et al. also reported that size distribution is time-dependent, with prolonged circulation resulting in a leftward shift in size distribution and thus smaller NBs.^31^

**Figure 2:**
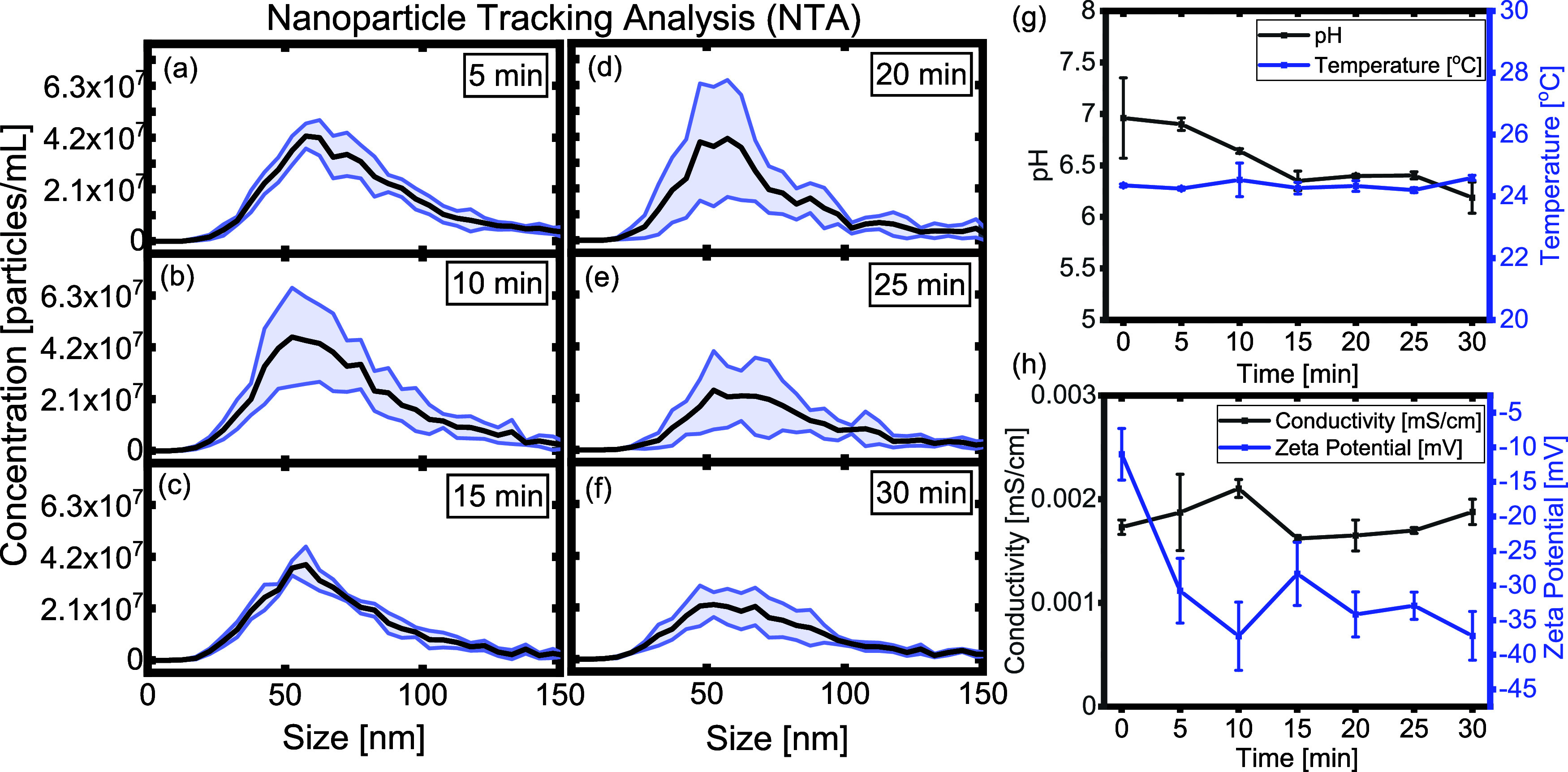
Nanoparticle tracking analysis (NTA) showing average particle size distribution of nanobubbles (NBs) in suspension created using 5-, 10-, 15-, 20-, 25-, and 30-min generation times (a–f). See Supporting Movie 1 for a video of the NBs moving under Brownian motion, recorded during the NTA measurements. (g) Evolution of pH and temperature over a 30 min generation. (h) Evolution of conductivity and zeta potential over a 30 min generation time.

The temperature of the NB suspension remained stable at around room temperature, with a starting temperature of 24.35 (±0.05) °C and a final temperature of 24.60 (±0.08) °C after 30 min of generation (Figure 2g), with no significant change observed over time (*p* > 0.05). The pH of the suspension significantly decreased (*p* < 0.01) over the generation time from a starting pH of 6.96 (±0.39) to a pH of 6.19 (±0.15). Similar decreases in pH have been reported in other works where nanobubbles were generated using air, and dissolution of CO_2_ contributes to acidification.^32^ However, some studies have reported increases in pH, highlighting that the pH behavior is highly dependent on gas composition and experimental conditions.^33,34^ It has been shown that a neutral pH like the one seen here is preferred for stability of the NBs.^35^ Additionally, it is suggested that NBs can produce ROS, which may also contribute to the observed slight decrease in pH.^22^ The overall conductivity of the NB suspension did not significantly change, with a starting conductivity of 0.0017 mS/cm increasing slightly to 0.0019 mS/cm (Figure 2h).

The zeta potential of the NB suspension showed a rapid decrease from −11.00 (±3.73) mV and then leveling off to a final value of −37.27 (±3.52) mV over the 30 min generation time, with a significant difference observed between the initial and final time points (*p* < 0.05). The zeta potential stabilized after the initial decrease, remaining relatively constant at longer generation times. Zeta potential is a measure of the magnitude of electrostatic repulsion/attraction between particles and is one of the fundamental parameters known to affect the stability of dispersed systems.^36–38^ As described in the literature, a zeta potential between ± 30 and ± 40 mV indicates that the suspension has moderate stability.^37^ Other studies have reported values of around −20 mV for zeta potential of air-generated NBs; however, these employed tap water for generation and/or have reported bubbles with larger average diameter than presented here, around 490 nm compared to less than 100 nm within this study.^39–41^ A link has been shown between bubble size and zeta potential, with the zeta potential increasing as the bubble size decreases.^41–43^ Therefore, as the NBs generated in this study had a mean diameter less than 100 nm, a zeta potential of −37 mV is to be expected. Despite the decrease in particle concentration at longer generation times, the zeta potential remained relatively stable. This lack of correlation between bubble number density and zeta potential has been reported previously, where zeta potential was shown to depend primarily on the surface charge of individual bubbles rather than their concentration.^33^

It has been suggested in the literature that NBs have the ability to generate hydroxyl radicals (^•^OH) upon collapse; therefore, a preliminary test was carried out using terephthalic acid (TA) as a probe.^44^ TA can be used as a probe as it reacts with hydroxyl radicals to form 2-hydroxyterephthalic acid (TAOH), which can be detected using fluorescence spectroscopy.^25^ Notably, all samples had an increased fluorescence intensity compared to the control (Figure S1). The fluorescence intensity peaked at 10 min NB generation time, 122.25% relative to the control (0 min). This preliminary test provided a basis for a more detailed investigation of ^•^OH generation, which was carried out using ESR spectroscopy. Based on the particle size, concentration, and characteristics, 10 min was chosen as the most suitable generation time for all further NB suspensions used in this study.

### Hydroxyl Radical Determination Using ESR

The properties of NBs have been widely characterized; however, the ability of NBs to generate ROS, particularly hydroxyl radicals, remains a controversial topic within the literature.^45^ Previous studies have suggested that ROS are generated upon bubble collapse; however, the mechanisms behind ROS generation and whether ROS are generated in sufficient quantities remains unclear.^45–48^ One hypothesis is that collapse results in high temperature and pressure (∼10 MPa and ∼5000 K, estimated based on models) leading to molecular dissolution of water into hydroxyl radicals (^•^OH) and hydrogen atoms (^•^H) (Figure 3a).^45^ Another is that ion accumulation and increase in interfacial electrical potential occur during collapse due to slower diffusion of the ion from the surface to the bulk phase compared to the rate of shrinkage. The sudden extinction of the gas/liquid interface creates a high energy environment where either water and/or oxygen molecules dissociate into radical species (Figure 3b).^45^

**Figure 3:**
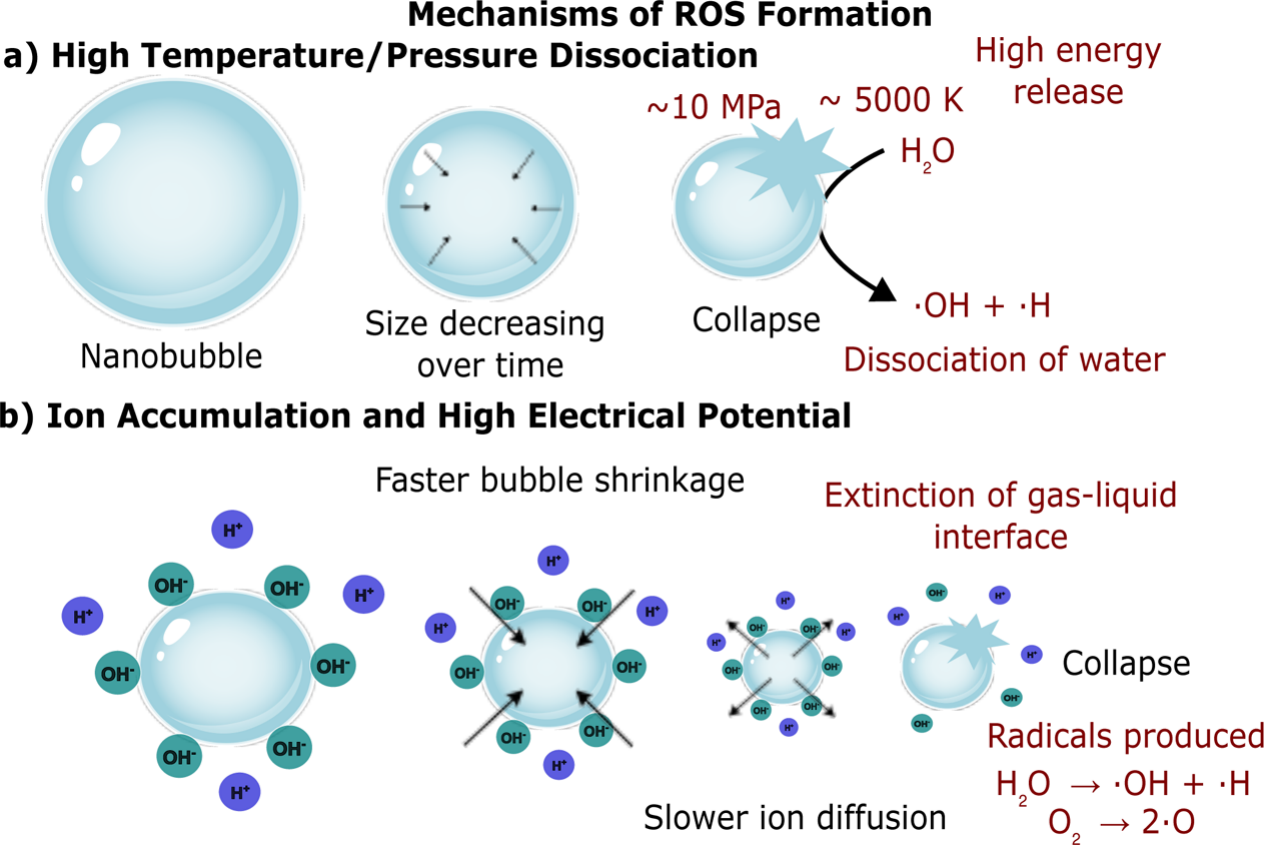
Proposed mechanisms of ROS formation from nanobubbles in water. (a) High temperature and pressure dissociation, and (b) ion accumulation and high electrical potential.

As preliminary tests with TA indicated the presence of ^•^OH radicals, ESR with the spin trapping agent DMPO was conducted to further evaluate the presence of radicals in the NB suspensions. Samples were mixed with DMPO, immediately frozen (to avoid absorption of microwaves in liquid suspensions), and then the ESR spectra measured continuously over 24 h at 250 K to monitor evolution of radicals. Measurements were performed at 250 K to stabilize spin adducts and improve spectral resolution; therefore, ESR results should be interpreted as qualitative and comparative indicators of radical generation rather than a direct representation of radical lifetimes or concentrations under ambient or biological conditions. A typical DMPO-OH quadruplet signal with an intensity ratio of 1:2:2:1 was observed, indicating the presence of hydroxyl radicals (Figure 4a).^49^ Other studies have also detected the typical DMPO-OH quadruplet signal within NB systems and are commonly used as evidence of ROS generation associated with NB formation and collapse.^50^ However, another study has contradicted this; for example, Chae et al. did not obtain any detectable ESR signal from ozone NBs.^45^ However, it must be noted that within the work conducted by Chae et al., measurements were taken 24 h after NB generation.^45^

**Figure 4:**
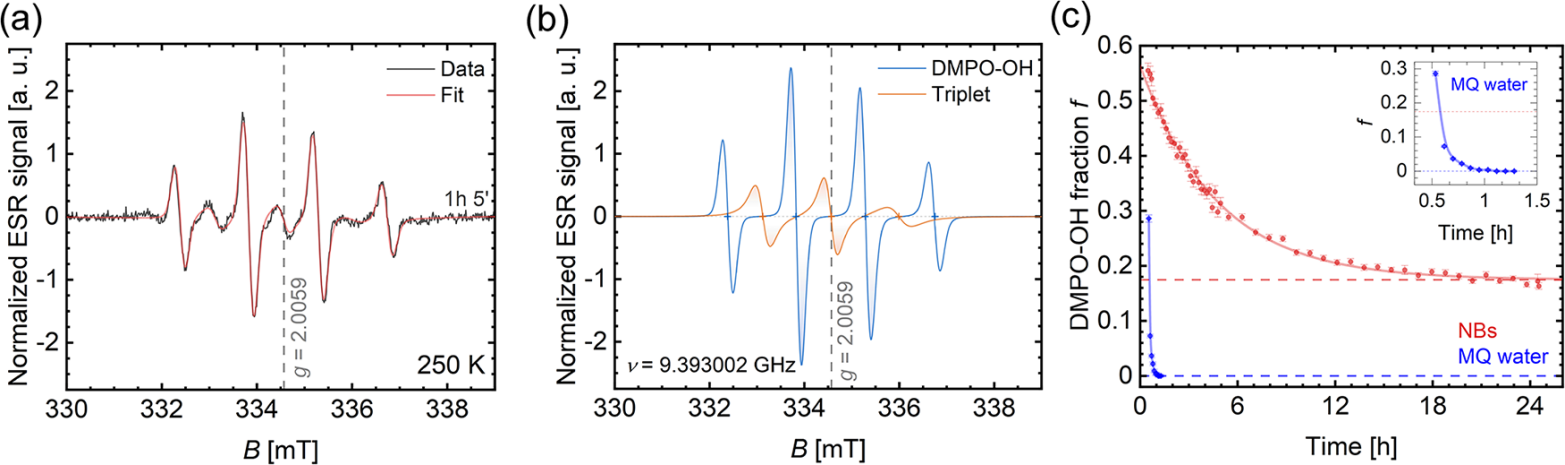
(a) Experimental ESR spectrum (black) and corresponding simulation (red) recorded at 250 K after 1 h, showing a quartet pattern characteristic of the DMPO-OH adduct. (b) Underlying, equal-concentration DMPO-OH (blue) and triplet (orange) signal contributions used in EasySpin simulations (see Materials and Methods section for details). (c) Time-dependent change in the DMPO-OH fraction (f) for NB water (red) and MQ water (blue). The red line shows an exponential fit of the NB time dependence with a characteristic time τ = (4.73 ± 0.15 h). The blue line is a guide to the eye. The inset highlights the rapid decay of *f* within the first 1.5 h for MQ water.

A triplet signal was also observed, which is suggested to arise from the decay of the DMPO-OH adduct, although its exact origin remains uncertain, with some studies attributing it to DMPOX or other oxidation products.^27^ The overall spectrum was decomposed into individual components using EasySpin simulations (Figure 4b). The blue line corresponds to the simulated DMPO-OH spectrum, and orange corresponds to the triplet signal. A simulated fit is also shown with good agreement between the data and the fit. By integration of the simulated components, the DMPO-OH fraction, *f*, was defined as the ratio of the DMPO-OH contribution to the total ESR signal intensity.

Due to the debate surrounding the generation of hydroxyl radicals and conflicting findings within the literature, a time-dependent change in *f* was explored over 24 h for NBs (red) and MQ water (blue) (Figure 4c). Immediately after preparation, the NBs show a high DMPO-OH fraction (*f* ≈ 0.5–0.6), indicating hydroxyl radicals are the dominant trapped species. Over the 24 h period, this fraction decays gradually and begins to level off around *f* ≈ 0.2. In contrast, MQ water shows a smaller starting DMPO-OH fraction (*f* ≈ 0.3), and this rapidly decreases to near the detection limit within 45 min (shown in the inset of Figure 4c). This rapid decay in MQ water is consistent with short-lived hydroxyl radicals and instability of the DMPO-OH adduct, resulting in a triplet-signal-giving state. Previous studies have shown the decay of the DMPO-OH adduct within tens of minutes, and an accompanied rise of the triplet signal has also been observed.^27,51^

In contrast, the much slower decay of the DMPO-OH quadruplet signal in NBs cannot solely be attributed to the higher initial radical concentration as this would not alter the decay time constant. Instead, it suggests differences in the frozen environment of NBs compared to MQ water that influence the stability and subsequent decay of the DMPO-OH adduct. Research has shown that rapid freezing of samples can decrease radical decay rates but also that spin adduct behavior is sensitive to the properties of the frozen medium.^52,53^ Regardless, the results show clear differences in the initial DMPO-OH signal intensity and decay rate between MQ water and NBs, therefore suggesting hydroxyl radical trapping as seen with other studies.^46,47,54^

### Interaction of NBs with E. coli and S. Aureus Biofilms

ESR analysis showed the presence of hydroxyl radicals; therefore, the interaction of NBs with single species biofilms of *E. coli* and *S. aureus* was explored to assess antimicrobial effects. The results were compared to MQ water to separate the effects caused by the NBs and those resulting solely from exposure to water, recognizing that nonphysiological, ion-free water can itself induce bacterial inactivation. Exposure to MQ water yielded a 1.04 (±0.45) log reduction in *E. coli* biofilm from 2.26 × 10^8^ to 2.23 × 10^7^ CFU/mL after a 64 min contact time (Figure 5a). This effect is likely attributable to a combination of nutrient deprivation, osmotic stress, and physical detachment of loosely adhered cells during treatment and recovery, rather than true bactericidal activity. Consequently, MQ water represents a conservative control accounting for nonspecific physical removal and handling effects. In contrast, NB treatment resulted in a 2.16 (±0.51) log reduction from 1.58 × 10^8^ to 1.65 × 10^6^ CFU/mL. A two-way ANOVA revealed a significant difference between NBs and MQ water [*F*(1, 112) = 48.96, *p* < 0.0001], and Tukey’s test showed NBs achieved significantly greater log reductions from 4 min onward, with effects increasing up to 64 min (*p* < 0.05).

**Figure 5:**
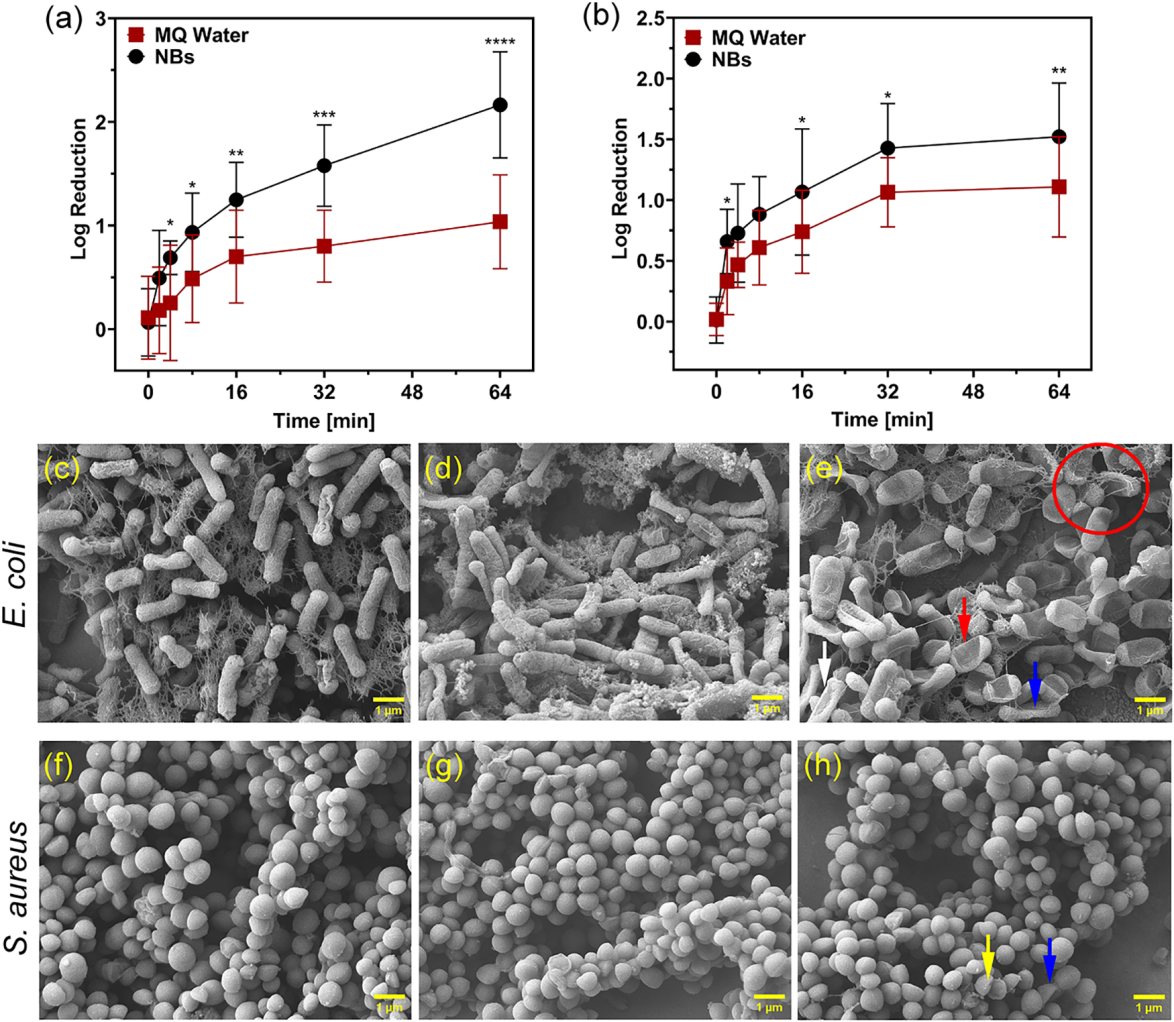
Log reduction in viable (a) *E. coli* and (b) *S. aureus* biofilms after Milli-Q water (MQ) and nanobubble (NB) treatment. Scanning electron microscopy (SEM) images depicting the morphological changes of *E. coli* (c) control, (d) MQ water, and (e) NB treated, and *S. aureus* (f) control, (g) MQ water, and (h) NB-treated biofilms on stainless steel coupons. Data are presented as mean ± SD (*n* = 9). Statistical significance between MQ water and NB treatments at each time point was determined using two-way ANOVA with Tukey’s post hoc test (*p < 0.05, **p < 0.01, ***p < 0.001, ****p < 0.0001).

For *S. aureus*, there was a 1.52 (±0.44) log reduction from 6.36 × 10^8^ to 1.98 × 10^7^ CFU/mL for NB treatment and a 1.11 (±0.41) log reduction for treatment with MQ water from 5.59 × 10^8^ to 5.07 × 10^7^ CFU/mL (Figure 5b). A two-way ANOVA revealed a significant difference between NBs and MQ water [F­(1, 112) = 22.22, *p* < 0.0001], and Tukey’s test showed NBs achieved significantly greater *S. aureus* log reductions at 2, 16, 32, and 64 min (*p* < 0.05). *S. aureus* proved to be more resistant to NB treatment than *E. coli*, likely due to *S. aureus* being a Gram-positive bacterium whereas *E. coli* is Gram-negative.^55^ Gram-positive bacteria are surrounded by a single thick peptidoglycan cell wall (80 nm), unlike Gram-negative species, which have a much thinner cell wall (∼8 nm thick) and an outer lipopolysaccharide membrane (1–3 nm thick).^55,56^

SEM was used to assess morphological changes to the cells and biofilm structure following NB treatment. The SEM images of control (untreated) *E. coli* biofilms on stainless steel show the typical rod-shaped cells with EPS surrounding them (Figure 5c). Figure 5d shows that exposure to MQ water did not result in much difference in the cell morphology, with only a few collapsed cells observed when compared to the untreated control. Interestingly, *E. coli* biofilms treated with NBs displayed a noticeable cell deformation, specifically collapse of the bacterial cells (red arrow) (Figure 5e). This collapse is consistent with disruption of cell envelope integrity or induction of mechanical or osmotic stress.^57^ The outer membrane of *E. coli* is filled with pore-forming proteins, which make it “leakier” than the cytoplasmic membrane and thus potentially easy for the NBs to pass through via simple diffusion.^58^ In addition, wrinkling of the cell surface (white arrow) and surface pitting (blue arrow) were observed. Notably, major membrane rupture or lysis was not observed, indicating that the cellular membranes remained largely intact. It can also be seen that there is disruption of the biofilm matrix compared to both the control and MQ water (red circle); however, this was not investigated further.

For the *S. aureus* biofilms, the typical cocci shape can be seen in the control (Figure 5f), while in both the MQ water (Figure 5g) and NB (Figure 5h) treated samples, cell fragmentation (yellow arrow) and pitting (blue arrow) can be observed. There was little obvious difference between the MQ water and NB treatments. These findings further highlight the difference in effects of the NBs against Gram-negative and Gram-positive bacteria. Many studies have shown Gram-positive bacteria to be more resistant to the activity of other nanoparticles.^59–61^

The effects of NBs on biofilm cell viability were further assessed using live/dead staining. Fluorescence microscopy images of *E. coli* and *S. aureus* biofilms are presented in Figure 6a and 6b, respectively, with green fluorescence indicating live cells and red fluorescence indicating dead cells (scale bars = 50 μm). In both *E. coli* and *S. aureus* biofilms, a clear increase in red fluorescence following NB treatment, corresponding to a higher proportion of compromised or dead cells was seen. However, there was still presence of green fluorescence, highlighting that the NB treatment was not capable of complete kill. This is consistent with CFU and SEM findings. Live/dead staining revealed a marked reduction in the live:dead cell ratio following NB treatment for both *E. coli* and *S. aureus* biofilms (Figure S2). For *E. coli*, the mean live:dead ratio decreased from 2.32 (±0.81) in the untreated control to 1.68 (±0.60) after exposure to MQ water, and further to 0.99 (±0.06) following NB treatment. Similarly, *S. aureus* biofilms showed a reduction from 1.52 (±0.51) (control) to 1.41 (±0.40) (MQ water) and 1.01 (±0.02) with NBs. Together, these findings indicate that NBs reduce biofilm viability in both species, with a stronger disruptive effect on *E. coli*.

**Figure 6:**
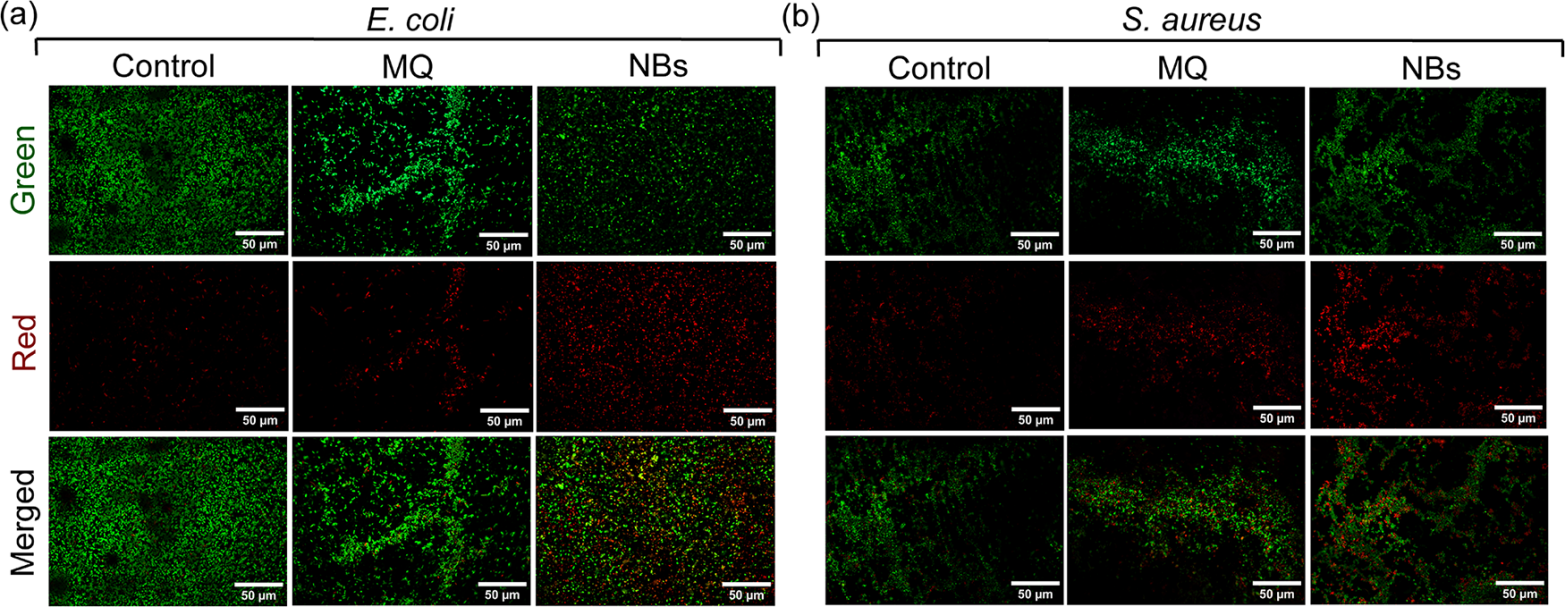
Fluorescence microscopy images of live/dead staining of biofilms formed by (a) *E. coli* and (b) *S. aureus* treated with Milli-Q water (MQ) and nanobubbles (NBs). Live cells are stained green (SYTO9), while dead cells are stained red (propidium iodide). The merged images display a mix of live and dead cells.

### Evaluation of Oxidative Stress

Further investigation was carried out to assess the contribution of any generated ROS to the antimicrobial effects of NBs. This was assessed by exploring oxidative stress through intracellular ROS and lipid peroxidation analysis within the biofilms. Intracellular ROS was measured via fluorescence spectroscopy (Figure 7a). The mean fluorescence intensity of *E. coli* treated with MQ water and NBs was 110.67% (±13.37) and 116.39% (±16.82) relative to the control, respectively. For *S. aureus*, the mean fluorescence intensity was 100.45% (±1.90) for MQ water and 109.69% (±10.73) for NBs, relative to the control. Although intracellular ROS levels were slightly elevated in NB-treated samples compared to MQ water, the differences were not statistically significant. These findings suggest that while NBs may induce a mild oxidative response, the ROS generated may not be at sufficient concentrations for intracellular oxidative stress to be the primary driver of antimicrobial activity under these conditions.^45^

**Figure 7:**
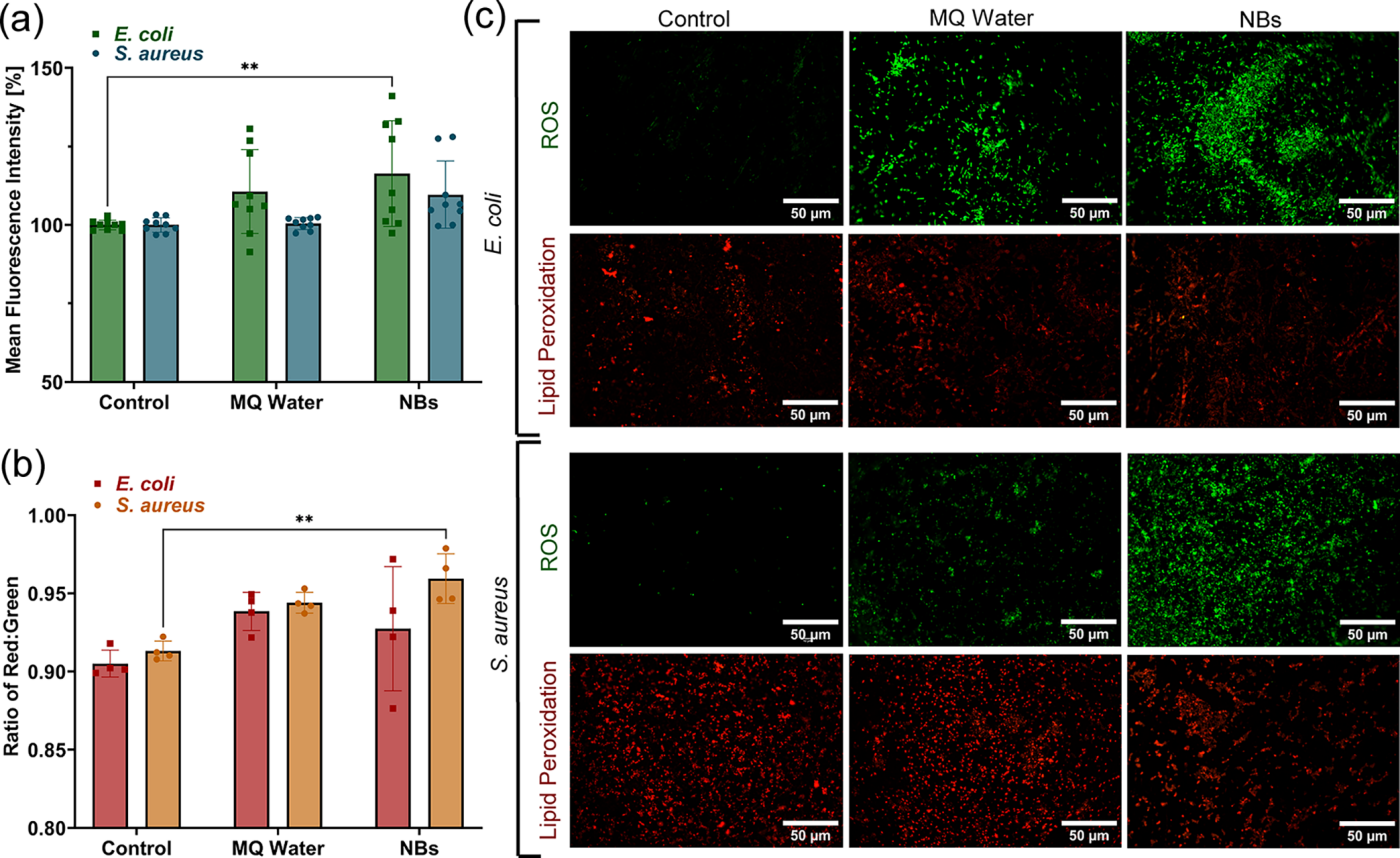
(a) Mean fluorescence intensity from intracellular ROS assay for *E. coli* and *S. aureus* biofilms exposed to Milli-Q water (MQ), and nanobubble (NB) treatments. Values are expressed relative to the control. (b) Lipid peroxidation ratio (red-to-green fluorescence) for biofilms under the same treatment conditions. (c) Representative fluorescence microscopy images showing ROS (green) and lipid peroxidation (red) in biofilms. Data are presented as mean ± SD. Statistical analysis: two-way ANOVA with Tukey’s multiple comparisons test, **p < 0.01.

Lipid peroxidation is a well-established consequence of oxidative stress, arising from the attack of ROS on membrane lipids, especially polyunsaturated fatty acids. Lipid peroxidation levels were measured in *E. coli* and *S. aureus* biofilms following NB treatment. In *E. coli*, the control group showed a value of 0.91 (±0.009), while MQ water and NB treatments resulted in values of 0.94 (±0.01) and 0.93 (±0.04), respectively. Similarly, in *S. aureus*, lipid peroxidation levels were 0.91 (±0.01) in the control, and 0.94 (±0.01) and 0.96 (±0.02) for MQ water and NB treatments, respectively. No significant differences were observed across treatments, although *S. aureus* treated with NBs showed a slightly higher ratio compared to the control (Figure 7b). The lack of detectable lipid peroxidation indicates that the observed cellular collapse is unlikely to be a result of oxidative damage to membrane lipids, therefore leaning to a more mechanical response.^57^

Figure 7c displays fluorescence images of ROS assays and lipid peroxidation in *E. coli* and *S. aureus* biofilms formed on stainless steel coupons, highlighting the presence of ROS in the treated samples compared to the control and very minor differences in lipid peroxidation. Intracellular nitric oxide levels were also measured, but these remained unchanged across all treatments (Figure S3). Collectively, these findings demonstrate that although hydroxyl radicals can be detected in NB suspensions using ESR, they do not translate into substantial intracellular oxidative stress or membrane lipid damage in biofilm cells. This disconnect indicates that ROS are unlikely to be the primary drivers of antimicrobial activity under the conditions studied and instead supports a predominantly nonoxidative, physical, or physicochemical mechanism of biofilm disruption. It is important to emphasize that ESR detection of hydroxyl radicals reflects radical formation within the bulk aqueous phase or at bubble interfaces, rather than direct exposure of biofilm-associated cells to these species.^45,51^ Hydroxyl radicals are extremely short-lived and react near their point of formation; therefore, ESR-detectable DMPO-OH adducts do not necessarily indicate biologically accessible ROS at the biofilm–cell interface.^51^ This distinction is critical for interpreting the relevance of physicochemical ROS measurements to biological outcomes.

### Long-Term Stability and Antimicrobial Efficacy of NBs

The long-term stability and antimicrobial activity of NB suspensions stored for up to 28 days was evaluated against *E. coli* and *S. aureus* biofilms. NTA demonstrated that the concentration of the 10 min NB suspension reduced from 5.66 × 10^8^ particles/mL to 4.57 × 10^8^ particles/mL after 28 days and the mean size decreased slightly from 84 to 81 nm (Figure 8a). In addition, the zeta potential decreased from around −37 mV to −26.07 (±3.32) mV (Table S2). These findings indicate that while some reduction in concentration and surface charge occurred over time, NBs remained present and largely stable for extended periods, supporting their potential for practical applications where prolonged storage may be required. This has also been reported in other studies with various types of NBs; however, up to 50–70% of bubbles can disappear after 50 days.^29,62^

**Figure 8:**
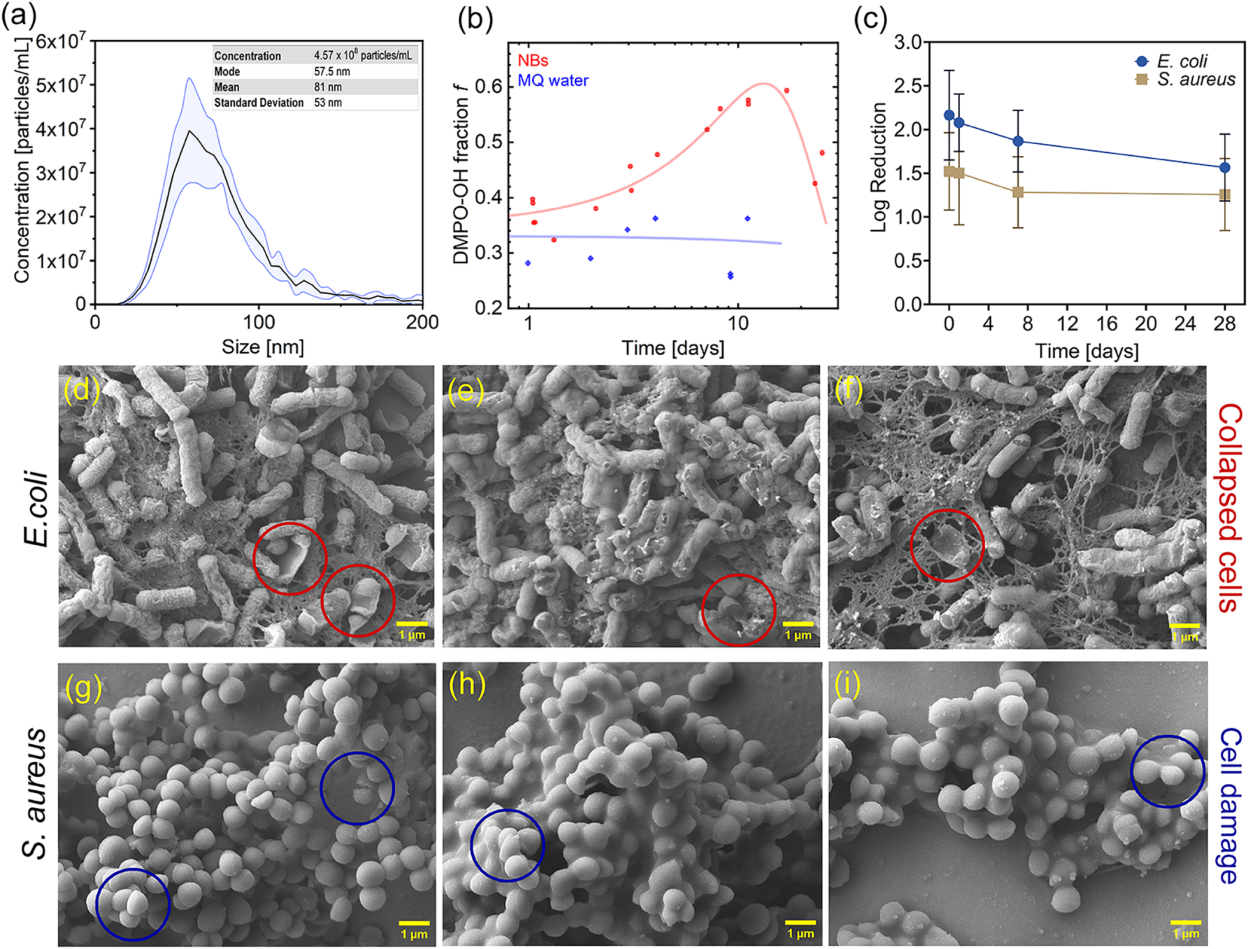
Long-term stability and antimicrobial efficacy. (a) Average particle size distribution of nanobubbles (NBs) stored for 28 days. (b) Long-term evolution of the DMPO-OH fraction (f) for NB water (red) and MQ water (blue). (c) Log reduction in viable cell count for *E. coli* (blue) and *S. aureus* (gold) after treatment with NBs stored for up to 28 days. (d–f) Scanning electron microscopy (SEM) images of *E. coli* biofilms treated with NBs stored for 1, 7, and 28 days, respectively, highlighting collapsed cells (red circles). (g–i) SEM images of *S. aureus* biofilms treated with NBs stored for 1, 7, and 28 days, respectively, showing visible cell damage (blue circles).

Investigation of the long-term hydroxyl radical activity in NBs (red) compared to MQ water (blue) was carried out (Figure 8b). Unlike previous continuous ESR measurements, long-term measurements were carried out by storing the samples for the indicated number of days, starting at 1 day, adding DMPO, freezing, and recording ESR spectra. While the 24 h measurements show a slow decay of the initially formed DMPO-OH adduct (*f* decreasing from ∼0.5–0.6 to ∼0.2), the long-term measurements reveal that hydroxyl radical activity within NB suspensions evolves over time with *f* increasing from ∼0.35–0.4 at 1 day to ∼0.6 after 14 days before decreasing at later time points. The delayed increase in *f* may reflect ongoing bubble shrinkage or collapse events that intermittently promote radical formation.^47^ In MQ water, *f* remains low and relatively constant throughout the measurement period. Differences in the ESR spectra between NBs and MQ water can be seen in Figure S4. Findings are consistent with Takahashi et al., who observed an ESR spectrum of the DMPO-OH adduct with NBs up to after 6 months of generation.^47^

As shown in Figure 8c, the antimicrobial activity of the NB suspensions reduced over the storage period. A two-way ANOVA revealed a significant effect of time on biofilm reduction (*F*(3,64) = 3.77, *p* = 0.0148), indicating a decrease in efficacy over the 28-day period. For *E. coli* biofilms, the log reduction in viable cell counts after NB treatment significantly decreased from 2.16 (±0.51) when NBs were freshly prepared to 1.57 (±0.38) after 28 days of storage. For *S. aureus* biofilms, the log reduction in viable cell counts after NB treatment reduced from 1.52 (±0.25) to 1.26 (±0.41) after 28 days of storage; however, this was not significant. For MQ water stored for the same time period, log reductions remained mostly consistent around 1 log reduction, with *E. coli* at 1.04 (±0.45) on day 0 and 1.16 (±0.42) on day 28, while *S. aureus* showed 1.11 (±0.41) on day 0 and 1.20 (±0.30) on day 28 (Figure S5). The structural damage to biofilms caused by NB treatment was further analyzed using SEM (Figure 8d–i). All stored NB suspensions still caused noticeable damage in the form of collapsed or damaged cells; however, the degree of damage appeared reduced, reflecting the decreased effectiveness of the older NB suspensions. Intracellular ROS levels were also assessed in response to treatment with stored NB suspensions (Figure S6). *E. coli* biofilms treated with fresh NBs showed mean fluorescence intensities of 116.39% (±16.82) relative to the control; however, this reduced to 102.89% (±5.05) at day 28. Similarly, *S. aureus* displayed 109.69% (±10.73) for fresh NBs reducing to 102.83% (±5.63) at day 28. Although the DMPO-OH signal showed persistent radical generation, antimicrobial activity against *E. coli* and *S. aureus* diminishes with storage time, indicating that ROS alone cannot explain antimicrobial efficacy and that short-lived ROS and/or physical NB effects are most likely more important.

### Conclusions

In conclusion, this study demonstrates that NBs can disrupt biofilms of *E. coli* and *S. aureus* on stainless steel, highlighting their potential as a physical disruption strategy. NBs generated for 10 min reached the highest stability and activity, achieving up to a 2.16 log reduction in *E. coli* and 1.52 log reduction in *S. aureus*, compared with only around 1 log reduction for MQ water alone. While ESR confirmed hydroxyl radical presence via DMPO-OH adduct formation in NB suspensions, intracellular oxidative stress and lipid peroxidation assays indicated that the observed antimicrobial effects are inconsistent with intracellular oxidative stress being the primary driver of biofilm inactivation under these conditions. This instead suggests mechanical or physicochemical interactions at the bubble–cell interface. Ultimately, the findings indicate that NBs could be a promising approach for biofilm disruption, offering long-lasting antimicrobial action without the use of environmentally hazardous precursors. While the NBs examined in this study did not achieve complete biofilm eradication, their ability to destabilize biofilm architecture and reduce viable cell numbers without reliance on chemical oxidation positions them as an attractive adjunct technology. In practical applications, NBs may be most effective when combined with low-dose chemical disinfectants, enzymatic treatments, or hydraulic flushing, where physical biofilm weakening can enhance downstream disinfection efficiency. Future work should extend this research to environmentally relevant, mixed-species biofilms derived from natural and engineered water systems and focus on elucidating the physicochemical mechanisms governing NB–biofilm interactions. A clearer understanding of these pathways will support optimization of NBs for sustainable disinfection and microbial control in wastewater treatment and water reuse applications.

## Supplementary Material





## Notes

EasySpin parameters, hydroxyl radical determination, biofilm assays, zeta potential data, and ROS/NO quantification (Tables and Figures) (PDF)
Brownian motion of NBs recorded using nanoparticle tracking analysis (NTA) (Movie S1) (AVI)
