# Supplementary material for: Physicochemical and Antimicrobial Characterization of Nanobubbles Reveals Physical Disruption is the Primary Mode of Biofilm Inactivation
Source: ACS ES T Water. 2026 Jun 1;6(6):3852–63. doi: 10.1021/acsestwater.6c00252 (PMC13270502; doi:10.1021/acsestwater.6c00252)
Supplement: Supplementary file 1 [file ew6c00252_si_001.pdf]

Physicochemical and Antimicrobial Characterization of Nanobubbles Reveals Physical Disruption is Primary Mode of Biofilm Inactivation.

Naomi Northage[1], Matjaž Gomilšek[1,2], Martina Modic[1], Damjan Vengust[1], Andrej Zorko[1,2], Uroš Cvelbar[1], James L. Walsh[1,3]

[1]Jožef Stefan Institute, Ljubljana SI-1000, Slovenia

[2]Faculty of Mathematics and Physics, University of Ljubljana, Ljubljana SI-1000, Slovenia.

[3]York Plasma Institute, School of Physics, Engineering & Technology, University of York, York, YO10 5DD, UK

Supplementary Table S1. *EasySpin parameters of the DMPO-OH and triplet signals at 250 K, assuming common eigenaxes for all anisotropic quantities (g-factors and hyperfine couplings).*

Parameter	DMPO-OH	Triplet
g-factor	[2.0058, 2.0063, 2.0056]	[2.0074, 2.0039, 2.0065]
Hyperfine couplings	aN = [1.58, 1.24, 1.59] mT<br>aβH = [1.45, 1.42, 1.40] mT	aN = [0.664, 1.69, 1.96] mT
Gaussian line broadening (peak-to-peak width)	0.204 mT	0.132 mT
Rotational correlation time	13.7 ns	9.38 ns

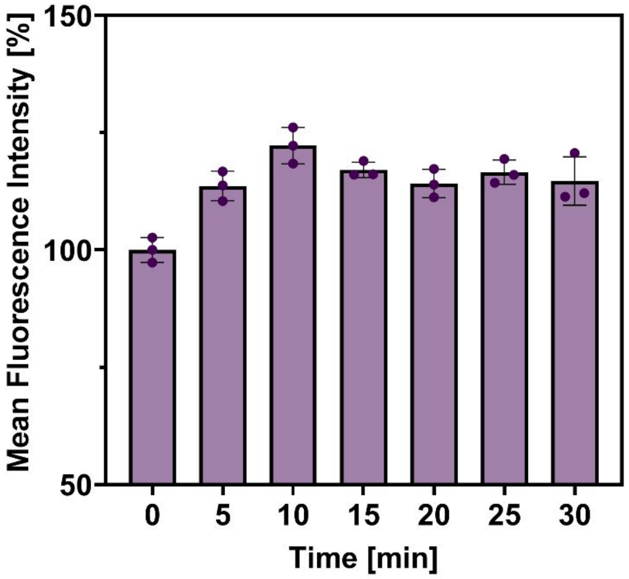

Supplementary Figure S1. *Determination of hydroxyl radicals (•OH) within nanobubbles (NBs) using terephthalic acid (TA) as a fluorescent probe. Data are presented as mean ± SD of fluorescence intensity, expressed as a percentage relative to the control (0 min).*

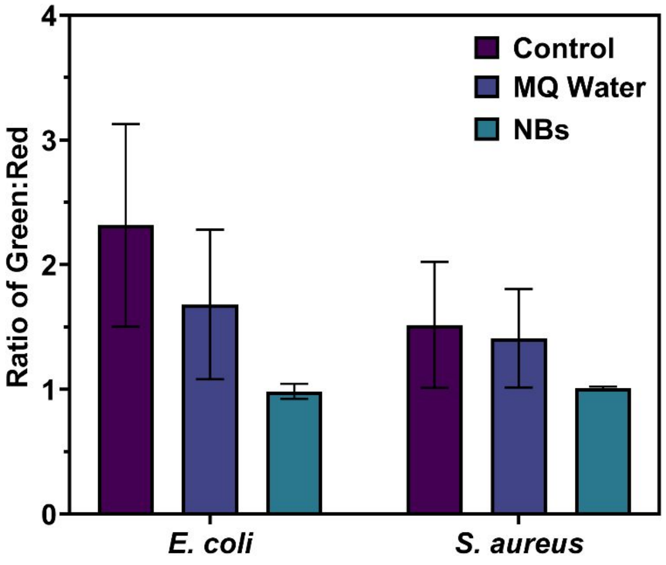

Supplementary Figure S2. *Fluorescence ratio of green to red for E. coli and S. aureus control, and MQ water and NB treated. Data are presented as mean ± SD.*

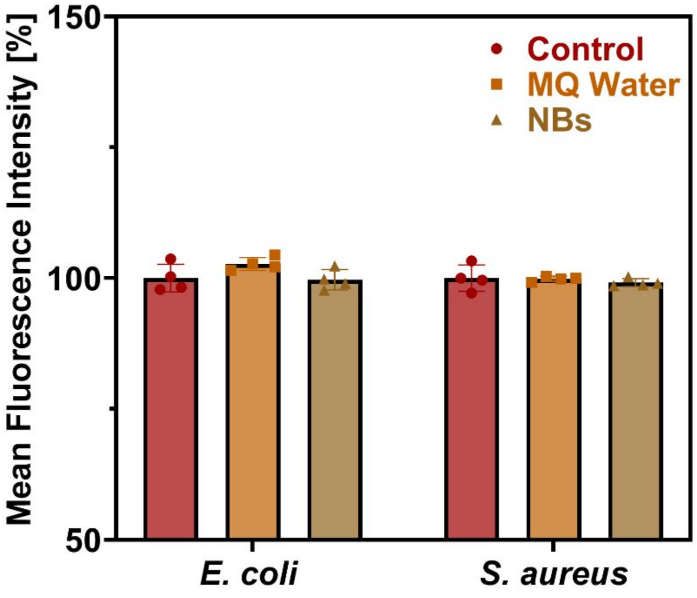

Supplementary Figure S3. *Quantification of intracellular nitric oxide (NO) levels in E. coli and S. aureus biofilms treated with MQ water and ultrafine bubbles (UFBs). Data are presented as mean ± SD fluorescence intensity, expressed as a percentage relative to the control.*

Supplementary Table S2. *Zeta potential measurements of nanobubble suspensions over a 28-day period. The table presents the zeta potential values recorded at specified time points, indicating the stability and surface charge characteristics of the nanobubbles during storage.*

	**Zeta Potential (mV)**	**Standard Deviation**
**Day 0**	-37.33	4.93
**Day 1**	-40.54	2.00
**Day 7**	-38.82	2.14
**Day 28**	-27.07	3.32

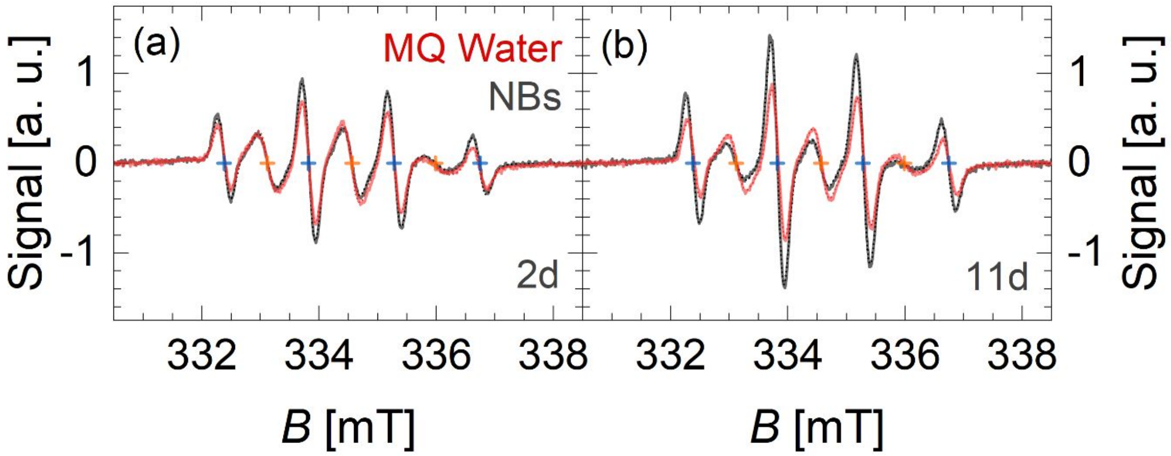

Supplementary Figure S4. *ESR spectra of MQ water (red) and NBs (black) measured after (a) 2 days and (b) 11 days of storage. Blue tick marks indicate the simulated hyperfine component positions. The spectra are normalized to highlight differences in line shape and signal intensity over the magnetic-field range 332–338 mT.*

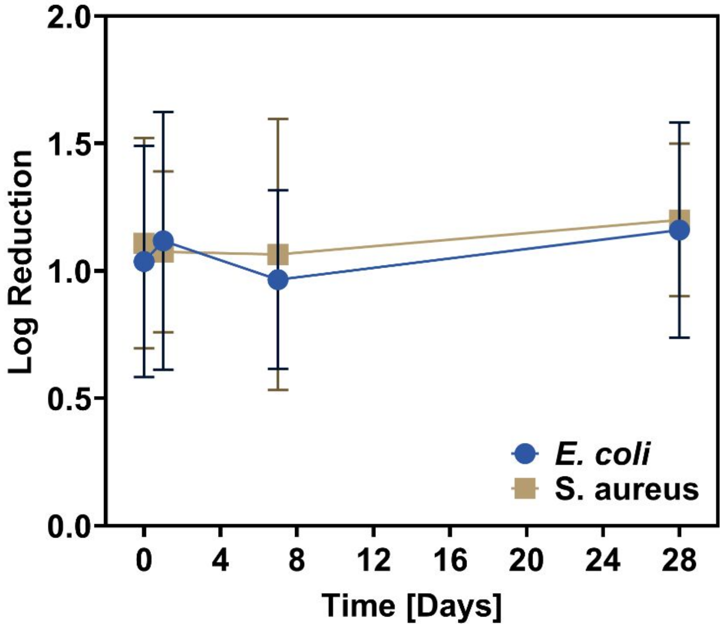

Supplementary Figure S5. *Log reduction of E. coli and S. aureus following exposure to MQ water stored for 1, 7, and 28 days. Data are presented as mean ± SD.*

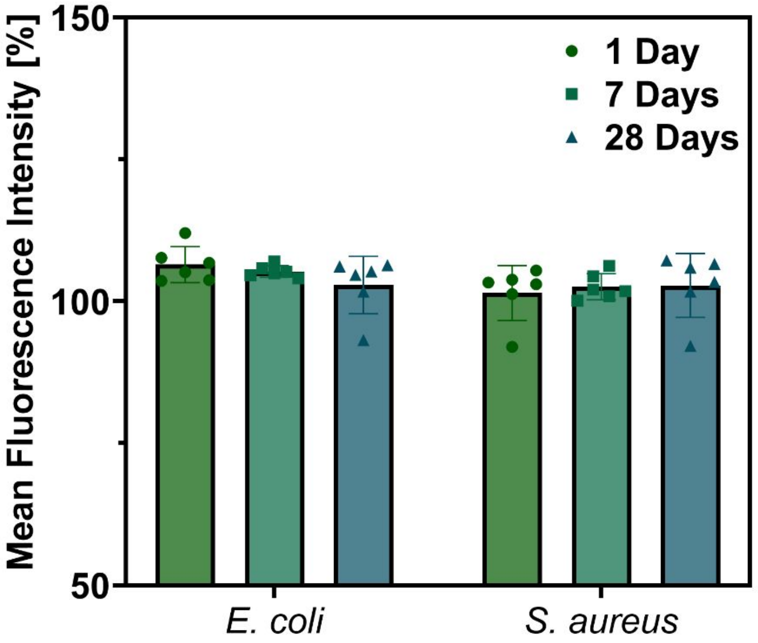

Supplementary Figure S6. *Intracellular ROS levels in E. coli and S. aureus biofilms exposed to nanobubbles (NBs) stored for 1, 7, and 28 days. Data are presented as mean ± SD fluorescence intensity, expressed as a percentage relative to the control.*
